# New Insight Into the Cardioprotective Effects of *Allium ursinum* L. Extract Against Myocardial Ischemia-Reperfusion Injury

**DOI:** 10.3389/fphys.2021.690696

**Published:** 2021-07-30

**Authors:** Marina Rankovic, Milos Krivokapic, Jovana Bradic, Anica Petkovic, Vladimir Zivkovic, Jasmina Sretenovic, Nevena Jeremic, Sergey Bolevich, Maria Kartashova, Jovana Jeremic, Stefani Bolevich, Vladimir Jakovljevic, Marina Tomovic

**Affiliations:** ^1^Department of Pharmacy, Faculty of Medical Sciences, University of Kragujevac, Kragujevac, Serbia; ^2^Faculty of Medicine, University of Montenegro, Krusevac, Montenegro; ^3^Department of Physiology, Faculty of Medical Sciences, University of Kragujevac, Kragujevac, Serbia; ^4^Department of Pharmacology, Sechenov First Moscow State Medical University, Moscow, Russia; ^5^Department of Human Pathology, Sechenov First Moscow State Medical University, Moscow, Russia; ^6^Department of Pathophysiology, Sechenov First Moscow State Medical University, Moscow, Russia

**Keywords:** *Allium ursinum* L., heart, ischemia/reperfusion injury, oxidative stress, rats

## Abstract

This study aimed to estimate the effects of increasing doses of *Allium ursinum* methanol extract on cardiac ischemia/reperfusion injury (I/R) with a special emphasis on the role of oxidative stress. Fifty rats were randomly divided into five groups (10 animals per group) depending on the applied treatment as follows: sham, rats who drank only tap water for 28 days and hearts were retrogradely perfused for 80 min without I/R injury, I/R, rats who drank only tap water for 28 days and hearts were exposed to *ex vivo* I/R injury and rats who consumed increasing doses of *A. ursinum* 125, 250, and 500 mg/kg for 28 days before I/R injury. Hearts from all rats were isolated and retrogradely perfused according to the Langendorff technique. Parameters of oxidative stress were spectrophotometrically measured in blood, coronary venous effluent, and heart tissue samples. Intake of wild garlic extract for 28 days significantly contributed to the recovery of cardiac function, which was reflected through preserved cardiac contractility, systolic function, and coronary vasodilatory response after ischemia. Also, wild garlic extract showed the potential to modulate the systemic redox balance and stood out as a powerful antioxidant. The highest dose led to the most efficient decrease in cardiac oxidative stress and improve recovery of myocardial function after I/R injury. We might conclude that wild garlic possesses a significant role in cardioprotection and strong antioxidant activity, which implicates the possibility of its use alone in the prevention or as adjuvant antioxidant therapy in cardiovascular diseases (CVD).

## Introduction

Acute myocardial infarction (AMI) and heart failure (HF) that often complicates this condition are among the leading causes of death and disability worldwide (Hausenloy et al., 2019). During AMI, coronary blood flow through arteries becomes severely limited or stopped, while tissue under the occlusion is deprived of oxygen and nutrients (Curran et al., 2019). Therefore, if the disrupted balance between myocardial oxygen demand and supply persists for a long time, the tissue necrosis occurs leading to the formation of a non-contractile fibrotic scar that reduces myocardial contractile force (Curran et al., 2019; Kusmic and L'Abbate, 2020). To date, timely and effective restoration of blood (reperfusion) has been driven as the gold standard strategy for the treatment of infracted myocardium. Although reperfusion provides great benefits through bringing fresh blood to the ischemic tissue, it also can cause irreversible myocardial damage termed as “ischemia/reperfusion (I/R) injury” (Kusmic and L'Abbate, 2020; Wang et al., 2020). Moreover, the reperfusion process is also known as a double-edged sword due to different associated pathologist. Restoration of blood flow to ischemic tissue also delivers blood-borne formed elements that in turn lead to increased oxidative stress by generating of ROS (reactive oxygen species) and bioavailability reduction of nitric oxide (NO) as an intracellular cardioprotective agent. These later cause exacerbation of parenchymal cell damage that finally induces secondary injury of tissue (Rout et al., 2020). The reperfusion damage includes a series of events such as reperfusion-induced arrhythmias, myocardial stunning, microvascular obstruction, and lethal myocardial reperfusion injury, of which only first two are reversible (Braunwald, 2015; Kusmic and L'Abbate, 2020). The final size and the severity of the tissue damaged mostly depends on cardiac or extra-cardiac factors including size of the occluded vessel, duration of reduced oxygen supply to ischemic tissue, neurohumoral activation, or inflammatory process (Wu et al., 2018). The pathogenesis of I/R injury consists of many mechanisms, while the exact underlying molecular mechanisms of lethal reperfusion injury are not fully known and remain a huge challenge for clinicians (Neri et al., 2017).

Given the fact that ischemic time is a critical determinant of cardiomyocytes death, ample evidence suggests the triggering of cardioprotective pathways during the first minute of reperfusion (Kusmic and L'Abbate, 2020). Among all studied therapeutic tools during I/R, cardioprotection induced by ischemic preconditioning represents one of the most powerful interventions that limits infarct size after I/R injury (Ferdinandy et al., 2014). Even in the mid-1980s, researchers reported that short episodes of ischemia followed by reperfusion could protect cardiomyocytes subsequently exposed to prolonged ischemia. This strategy named ischemic preconditioning, not only provided limited infarct size but also reduced incidence and duration of arrhythmic events and ventricular tachycardia and fibrillation (Neri et al., 2017). Following the beneficial effects of this strategy, it became clear that non-ischemic stimulus including pharmacological agents applied prior to the onset of myocardial ischemia could mimic cardioprotection induced by ischemic preconditioning (Li et al., 2015). Numerous data are indicating the powerful effects of medical plants enriched with polyphenols in preventing harmful effects of ischemic disease. The active compounds of plants exhibit multiple beneficial effects including anti-inflammatory, anti-apoptotic, and anti-cancer properties, but plant extracts are deemed as promising cardioprotective agents for decreasing ROS production in ischemic conditions (Li et al., 2017; Sedighi et al., 2019). In that sense, there is a continuing effort to discover cardioprotective compounds of natural origin which would alleviate the consequences of I/R injury on myocardial function.

*Allium ursinum* L. (synonyms: wild garlic, ramson, or bear garlic) is a perennial herbaceous plant belonging to the Alliacee family and has been used for centuries in traditional medicine as a prophylactic and therapeutic agent (Sobolewska et al., 2015). A growing interest in the usage of this plant species as a dietary supplement and food indicates the need to further investigate its therapeutic benefits. The high efficacy of *A. ursinum* for the prevention and treatment of cardiovascular system diseases has been documented, and it is based on the potential to lower blood pressure, insulin concentration, total cholesterol level, and an exert antiaggregatory effect (Sobolewska et al., 2015). Since this plant possesses a significantly higher level of ajoen compared to common garlic (*Allium sativum*) and 20-times the level of adenosine, wild garlic exerts powerful effects on the cardiovascular system. Sulfoxide-derived components of garlic are a major contributor to the antioxidant capacity of garlic extracts that scavenge free radicals and preserve tissue damage. Moreover, this plant inhibits activity of the angiotensin-converting enzyme and, thus, decreases systolic blood pressure but also reduces arrhythmias and the size of the ischemic zones (Rietz et al., 1993; Bombicz et al., 2016). Phytochemical investigations of *A. ursinum* revealed the presence of sulfur and phenolic compounds as the most characteristic constituents, which might be efficient in reaching cardioprotection (Sobolewska et al., 2015). Therefore, we supposed that *A. ursinum* extract might be involved in the mitigation of deleterious effects of I/R injury on functional properties of the heart.

Based on all the above mentioned data, this study aimed to estimate the effects of chronic the application of increasing doses of *A. ursinum* methanol extract (AUE) on myocardial function and coronary circulation under I/R injury with a special emphasis on the role of cardiac oxidative stress.

## Materials and Methods

### Ethical Approval

This investigation was conducted in the laboratory for cardiovascular physiology of the Faculty of Medical Sciences, University of Kragujevac, Serbia. The study protocol was approved by the Ethical Committee for the welfare of experimental animals of the Faculty of Medical Sciences, University of Kragujevac, Serbia. All experiments were performed according to EU Directive for the welfare of laboratory animals (86/609/EEC) and principles of Good Laboratory Practice (GLP).

### Plant Material and Extract Preparation

The whole plant *A. ursinnum* was collected in May 2018 on *Mt Bukulja (GPS coordinates: 40*°*17*′*55*″ *N and 20*°*31*′*45*^′′^ *E)*. Identification and classification of the plant material were performed at the Department of Biology, Faculty of Natural Sciences, University of Kragujevac and at the Institute of Botany and Botanical garden “Jevremovac,” University of Belgrade. Voucher specimens are deposited in the Herbarium of the Institute of Botany and Botanical garden “Jevremovac” with number 17417. The collected material was dried under the shade and powdered (sieve 0.75). The methanol extract was prepared by extracting 100 g of aerial part of the plant with 500 ml of methanol by heat reflux extraction, at the temperature of 90°C, in 2 h (Hijazi et al., 2015). The mixture was filtered through filter paper (Whatman, No. 1). The dry extract was obtained by evaporation under reduced pressure (RV05 basic IKA, Germany). The residue (6.7 g) was stored in a dark glass bottle at +4°C for further processing. To feed the animals, AUE was daily dissolved in the water just before administered to experimental animals.

### Animals and Experimental Design

The present study was carried out on 50 male *Wistar albino* rats (8 weeks old, bodyweight 200 ± 50 g, 10 rats per group). The animals consumed commercial rat food (20% protein rat food, Veterinary Institute Subotica, Serbia), and they were housed under controlled environmental conditions, at room temperature (23 ± 2°C) with an established photoperiod of 12-h light/day. The rats had free access to food and tap water, *ad libitum*. Rats were randomly divided into five groups depending on the applied treatment.

- Sham, rats who drank only tap water for 28 days and after sacrificing, their hearts were retrogradely perfused for 80 min without I/R injury;
- I/R, rats who drank only tap water for 28 days and after sacrificing, their hearts were exposed to *ex vivo* I/R injury;
- 125 AUE, rats who drank tap water containing 125 mg/kg of methanol extract of *A. ursinum* for 28 days before *ex vivo* I/R injury;
- 250 AUE, rats who drank tap water containing 250 mg/kg of methanol extract of *A. ursinum* for 28 days before *ex vivo* I/R injury;
- 500 AUE, rats who drank tap water containing 500 mg/kg of methanol extract of *A. ursinum* for 28 days before *ex vivo* I/R injury.

The hearts of male *Wistar albino* rats were excised and perfused on a Langendorff apparatus (Experimetria Ltd., 1062 Budapest, Hungary) following treatments. After a short-term ketamine/xylazine narcosis, animals were killed by cervical dislocation (Schedule 1 of the Animals/Scientific Procedures, Act 1986, UK) and premedicated with heparin as an anticoagulant. After emergency thoracotomy and rapid cardiac arrest by superfusion with ice-cold isotonic saline, the aortas were rapidly excised, cannulated, and retrogradely perfused under a constant perfusion pressure (CPP = 70 cmH_2_O). Krebs-Henseleit buffer was used for retrograde perfusion (in mmol/l: NaCl 118, KCl 4.7, ${\text{CaCl}}_{2}^{.}$2H_2_O 2.5, ${\text{MgSO}}_{4}^{.}$7H_2_O 1.7, NaHCO_3_ 25, KH_2_PO_4_ 1.2, glucose 11, and pyruvate 2). The buffer was balanced with 95% O_2_ and 5% CO_2_, with a pH of 7.4 and a temperature of 37°C. Immediately after the restoration of a normal heart rhythm, a sensor (transducer BS473-0184, Experimetria Ltd., Budapest, Hungary) was inserted into the left ventricle for continuous monitoring of cardiac function through a created entrance to the left atrium of the heart and damaged mitral valve. The following parameters of myocardial function have been continuously measured: maximum rate of pressure development in the left ventricle (dp/dt max), the minimum rate of pressure development in the left ventricle (dp/dt min), systolic left ventricular pressure (SLVP), diastolic left ventricular pressure (DLVP), and heart rate (HR). Coronary flow (CF) was measured flow metrically.

In all groups, isolated hearts were stabilized within 30 min when all cardiodynamic parameters and CF were measured (marked as S point). After the stabilization period, hearts from I/R and AUE treated groups were exposed to 20 min ischemia followed by 30 min reperfusion, while hearts from the sham group were perfused for 80 min without intervention. In addition to measurements in the stabilization (S) period, cardiodynamic parameters and CF were collected during the reperfusion period in intervals of 5 min from hearts exposed to the I/R protocols, and from 50th to the 80th min in the sham group (points 1–7).

### Oxidative Stress Markers and Antioxidant Enzyme Determination

After sacrificing the animals, blood samples for biochemical analysis were collected from a jugular vein to test the systemic redox state. Moreover, coronary venous effluent was collected in S and 1–7 time points, while hearts were collected after the accomplishment of protocols on the Langendorff apparatus.

Plasma samples and erythrocytes were separated *via* centrifugation of heparinized venous blood. In plasma samples and coronary venous effluent, we measured the concentration of pro-oxidative markers such as the index of lipid peroxidation, measured as thiobarbituric acid-reactive substances (TBARS), nitrites (NO_2_^−^), superoxide anion radical (O_2_^−^), and hydrogen peroxide (H_2_O_2_). In the lysate, we determined the activity of non-enzymatic antioxidants, including reduced glutathione (GSH) and the activity of the enzymatic defense system by evaluating the catalase (CAT) and superoxide dismutase (SOD) levels, while in heart tissue homogenates, we determined CAT and SOD activity. Heart tissue homogenates were prepared according to a previously published method (Bradic et al., 2020). All mentioned biochemical parameters of oxidative stress were determined spectrophotometrically (*Shimadzu UV-1800UV-VIS spectrophotometer, Japan*).

#### TBARS Determination (Index of Lipid Peroxidation)

The degree of lipid peroxidation in the coronary venous effluent and plasma samples was estimated by measuring TBARS, using 1% thiobarbituric acid in 0.05 NaOH, which was incubated with the coronary effluent at 100°C for 15 min and measured at 530 nm. Krebs-Henseleit solution was used as a blank probe (Bradic et al., 2020).

#### NO_2_^–^ Determination

Nitric oxide decomposes rapidly to form stable nitrite/nitrate products. The nitrite level (NO_2_^−^) was measured and used as an index of NO production, using Griess's reagent. A total of 0.5 ml of samples was precipitated with 200 μl of 30% sulpho-salicylic acid, vortexed for 30 min, and centrifuged at ×3,000 g. Equal volumes of the supernatant and Griess's reagent, containing 1% sulphanilamide in 5% phosphoric acid/0.1% naphthalene ethylenediamine-dihydrochloride were added and incubated for 10 min in the dark and measured at 543 nm. The nitrite levels were calculated using sodium nitrite as the standard (Bradic et al., 2020).

#### O_2_^–^ Determination

The level of the O_2_^−^ was measured *via* a nitro blue tetrazolium (NBT) reaction in TRIS buffer with coronary venous effluent or plasma samples, at 530 nm. Krebs-Henseleit solution was used as a blank probe (Bradic et al., 2020).

#### H_2_O_2_ Determination

The measurement of the level of H_2_O_2_ was based on the oxidation of phenol red by H_2_O_2_ in a reaction catalyzed by horseradish peroxidase (HRPO) (Bradic et al., 2020). Two hundred microliters of samples was precipitated using 800 ml of freshly prepared phenol red solution; 10 μl of (1:20) HRPO (made extempore) were subsequently added. For the blank probe, an adequate volume of Krebs-Henseleit solution was used instead of coronary venous effluent. The level of H_2_O_2_ was measured at 610 nm.

#### Determination of Markers of Antioxidant Defense

For SOD determination, samples that involve lysate and homogenate of heart tissue were mixed with carbonate buffer, and then epinephrine was added. Detection of SOD was performed at 470 nm. For CAT determination, CAT buffer, prepared sample, and 10 mM H_2_O_2_ were used. Detection was performed at 360 nm. The level of reduced glutathione was determined based on GSH oxidation with 5.5-dithio-bis-6.2-nitrobenzoic acid in erythrocyte samples. Detection was performed at 420 nm (Bradic et al., 2020).

### Histological Analysis

The isolated rat hearts were halved and the left and right ventricle wall was fully exposed. The heart samples were fixed in 4% neutral formalin, dehydrated in ethanol, cleared in xylene, embedded in Histowax^®^ (Histolab Product AB, Göteborg, Sweden), and processed for further histological analysis. Tissue sections, the thickness of 5 μm were stained with H/E for the visualization of tissue structures. From each half of the heart, 100 serial tissue sections were made. Analysis was performed on 10 tissue sections for each specimen. Images of tissue sections were captured with a digital camera attached to the Olympus BX51 microscope on magnification ×40 (Sretenovic et al., 2016). Criteria for histological analysis involved: presence (+) or absence (–) of degenerative changes, dilated interstitium, and hypercellularity. To estimate the intensity of changes, following score was used: (–) no morphological changes, (+) mild (<10% per cross-section), (++) moderate (<20% per cross-section), and (+ + +) severe (<30% per cross-section).

### Statistical Analysis

IBM SPSS Statistics 20.0 for Windows was used for statistical analysis of data. Three measured points were statistically analyzed: the first point was S, second was the first and the last point of 30 min reperfusion period (points 1 and 7) for I/R and treated groups and 50th and 80th min for the sham group. Values were expressed as mean ± SE. The distribution of data was checked by Shapiro–Wilk test. Data generated from time-course measurements (changes in parameters of *ex vivo* cardiac function and pro-oxidants in coronary venous effluent) were analyzed using two-way ANOVA and the *post-hoc* Bonferroni test for multiple comparisons. Additionally, other data (comparison between systemic redox markers and tissue enzymes activities) were analyzed using one-way ANOVA and the *post-hoc* Bonferroni test for multiple comparisons. Values of *p* < 0.05 were considered to be statistically significant, while values of *p* < 0.01 were considered to be highly statistically significant.

## Results

### The Effects of *A. ursinum* Extract on *ex vivo* Cardiac Function

#### Maximum Rate of Left Ventricular Pressure Development (dp/dt Max)

**Figure 1:**
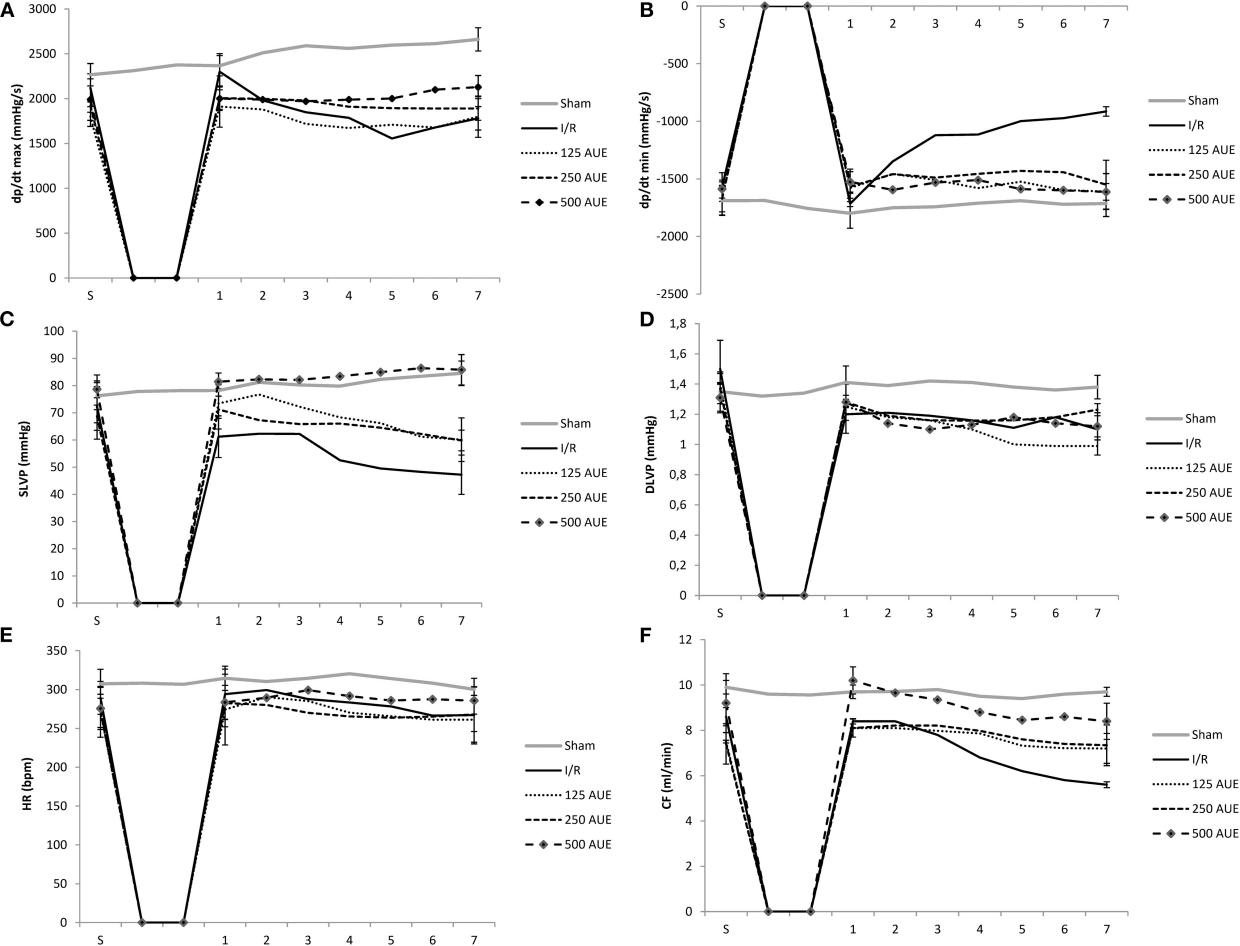
Effects of consumption of increasing doses of *Allium ursinum* methanol extract (AUE) on the value of maximum rate of pressure development in the left ventricle (dp/dt max) **(A)**, minimum rate of pressure development in the left ventricle (dp/dt min) (**B**), systolic left ventricular pressure (SLVP) **(C)**, diastolic left ventricular pressure (DLVP) **(D)**, heart rate (HR) **(E)**, coronary flow (CF) **(F)** (*n* = 50, 10 animals per group). Values are shown as mean ± SE.

Significantly higher dp/dt max values in point 1 and the last point 7 were noticed in the sham group compared to other groups. Moreover, in the I/R group, a significant increase in dp/dt max value was observed during the first moment of reperfusion compared to the stabilization period and the last moment of reperfusion (point 7). However, markedly higher values of dp/dt max were found in groups treated with AUE compared to I/R rats in the reperfusion period with the exception in the first point of the observed period. Additionally, the most prominent increase in dp/dt max was observed in the group of rats on the highest dose treatment compared to the other two applied doses of AUE (Figure 1 and Table 1).

**Table 1:** Cardiodynamic parameters within and between groups.

**Groups/**	**dp/dt max (mmHg)**	**dp/dt min (mmHg)**	**SLVP (mmHg)**
**parameters**			
**Sham**			
S	2267.1 ± 124.5	−1689.2 ± 124.2	76.2 ± 3.4
1	2367.2 ± 113.4^f^^−^^i^	−1798.7 ± 130^g^^−^^i^	78.2 ± 2.1^f^^,^^i^
7	2661.3 ± 130^f^^−^^i^	−1713.4 ± 113.4^f^^,^^g^	84.5 ± 4.5^f^^−^^h^
**I/R**			
S	2120.8 ± 157.16	−1571.26 ± 110.3	72.3 ± 4.5
1	2300.8 ± 201.6^f^	−1715.24 ± 53.4	61.2 ± 6.5^f^
7	1780.7 ± 130.5^f^	−914.8 ± 30.2^a^^,^^b^^,^^f^	47.2 ± 6.8^f^
**AUE125**			
S	1800.2 ± 110.3^*^	−1671.2 ± 18.2	72.1 ± 10.88
1	1912.3 ± 230^*^^g^	−1578.3 ± 78.5^g^	73.4 ± 5.2
7	1879.2 ± 108.2^c^^,^^d^^,^^g^	−1456.4 ± 57.8^*^^g^	60.1 ± 7.8^a^^,^^b^^,^^d^^,^^g^^*^
**AUE250**			
S	1920.5 ± 72.8	−1671.8 ± 209.1	68.7 ± 2.4
1	2005.6 ± 124.5^h^	−1567.6 ± 112.3^h^	71.2 ± 7.2^*^
7	1890.5 ± 132.3^h^	−1550.2 ± 131.2^*^	59.8 ± 3.8^e^^,^^h^^*^
**AUE500**			
S	1989.2 ± 231.4	−1588.3 ± 78.4	78.6 ± 3.1
1	2001.3 ± 132.3^*^^i^	−1526.5 ± 88.4^*^^i^	81.4 ± 3.2^i^^*^
7	2129.3 ± 129.3^a^^,^^d^^,^^i^^*^	−1612.95 ± 72.3^*^	85.8 ± 5.6^d^^,^^e^^*^
	**DLVP (mmHg)**	**HR (bpm)**	**CF (mL/min)**
**Sham**			
S	1.35 ± 0.13	307.4 ± 18.7	9.9 ± 0.3^f^^−^^h^
1	1.41 ± 0.11^f^^−^^i^	314.5 ± 15.6^f^^−^^i^	9.7 ± 0.3^f^^−^^h^
7	1.38 ± 0.08^f^^−^^i^	300.2 ± 14.3^f^^−^^h^	9.7 ± 0.2^f^^−^^h^
**I/R**			
S	1.5 ± 0.18	289.2 ± 21.2	8.6 ± 0.4^f^
1	1.2 ± 0.12^f^	294 ± 32.3^f^	8.4 ± 0.12^f^
7	1.1 ± 0.17^f^	267.1 ± 21.2^f^	5.6 ± 0.13^f^
**AUE125**			
S	1.4 ± 0.01	271.3 ± 29.7	7.5 ± 0.08^g^
1	1.25 ± 0.02^g^	274.2 ± 41.5^g^	8.1 ± 0.12^g^
7	0.99 ± 0.04^a^^−^c^c^^,^^g^^*^	261.2 ± 29.6^g^	7.2 ± 0.7^g^^*^
**AUE250**			
S	1.37 ± 0.1	272.5 ± 21.4	7.4 ± 0.89^h^
1	1.28 ± 0.01^h^	282.6 ± 30.6^h^	8.1 ± 0.4^h^
7	1.23 ± 0.04^c^^,^^e^^,^^h^	267.9 ± 35.8^h^	7.34 ± 0.9^e^^,^^h^^*^
**AUE500**			
S	1.31 ± 0.1	275.6 ± 27.1	9.2 ± 1.3
1	1.28 ± 0.12^i^	283.4 ± 22.1^i^	10.2 ± 0.6^*^
7	1.12 ± 0.09^a^^,^^e^^,^^i^	285.7 ± 17.4^*^	8.4 ± 0.8^b^^,^^e^^*^

*Mean values ± SE in three points of interest. S, stabilization; 1, first minute of reperfusion; 7, 30th min of reperfusion*;

* *Statistically significant difference at the level of p < 0.05 between the group I/R and Allium ursinum methanol extract (AUE) groups at the point of interest*;

a *Statistically significant difference at the level of p < 0.05 in relation to point S within the group*;

b *Statistically significant difference at the level of p < 0.05 in relation to the point 1 within the group*;

c *Statistically significant difference at the level of p < 0.05 between AUE125 and AUE250*;

d *Statistically significant difference at the level of p < 0.05 between AUE125 and AUE500*;

e *Statistically significant difference at the level of p < 0.05 between AUE250 and AUE500*;

f *Statistically significant difference at the level of p < 0.05 between sham and I/R*;

g *Statistically significant difference at the level of p < 0.05 between sham and AUE125*;

h *Statistically significant difference at the level of p < 0.05 between sham and 250AUE*;

i *Statistically significant difference at the level of p < 0.05 between sham and AUE500*.

#### Minimum Rate of Left Ventricular Pressure Development (dp/dt Min)

Comparing the value of dp/dt min between groups treated with AUE and rats from I/R and sham groups, it was observed a statistically significant increase in this parameter in point 7 in groups of rats treated with different doses of extract. However, dp/dt min had similar values after the application of all three doses of the extract (Figure 1 and Table 1).

#### Systolic Blood Pressure in the Left Ventricle

Significantly higher values of SLVP during points 1 and 7 were observed in the sham group comparing to the I/R and rats treated with lower doses of AUE. Additionally, treatment with the highest AUE led to an improved value of this parameter compared to the sham group. In the I/R group, it was observed a drop in the SLVP value during the reperfusion period compared to stabilization. Moreover, the application of different doses of AUE led to a significant increase in the value of SLVP at all points of interest compared to the I/R group. However, comparing the influence of different extract doses on the value of this parameter, we observed the most significant increase in SLVP in rats treated with the highest dose of *A. ursinum* compared to two others applied doses (Figure 1 and Table 1).

#### Diastolic Left Ventricular Pressure

During the period of stabilization, there were no statistical differences in the values of this parameter between examined groups. However, during points 1 and 7, significantly higher DLVP values were noticed in the sham group compared to the others. Comparing the effects of different doses of AUE, values of DLVP did not vary significantly during observed points of interest, except for significantly higher DLVP values after administration of the mean dose relative to the highest and lowest at the last point 7 and highest relative to the lowest dose (Figure 1 and Table 1).

#### Heart Rate

The highest HR value was observed in sham rats during all points of interest in comparison to the animals that were exposed to I/R injury. A statistically significant increase in HR was observed after administration of the highest dose compared to others in the last minute of the following period point 7 and compared to the I/R group (Figure 1 and Table 1).

#### Coronary Flow

Rats treated with AUE reached significantly higher values of CF at the end of the following period compared to the I/R group. However, administration of the highest dose of *A. ursinum* led to the most prominent increase in CF value in all reperfusion periods as well as before ischemia compared to untreated rats and two other experimental groups. However, the highest CF value was observed in the sham group (Figure 1 and Table 1).

### Effects of *A. ursinum* Extract on Cardiac Redox State

#### Index of Lipid Peroxidation (Measured as TBARS)

**Figure 2:**
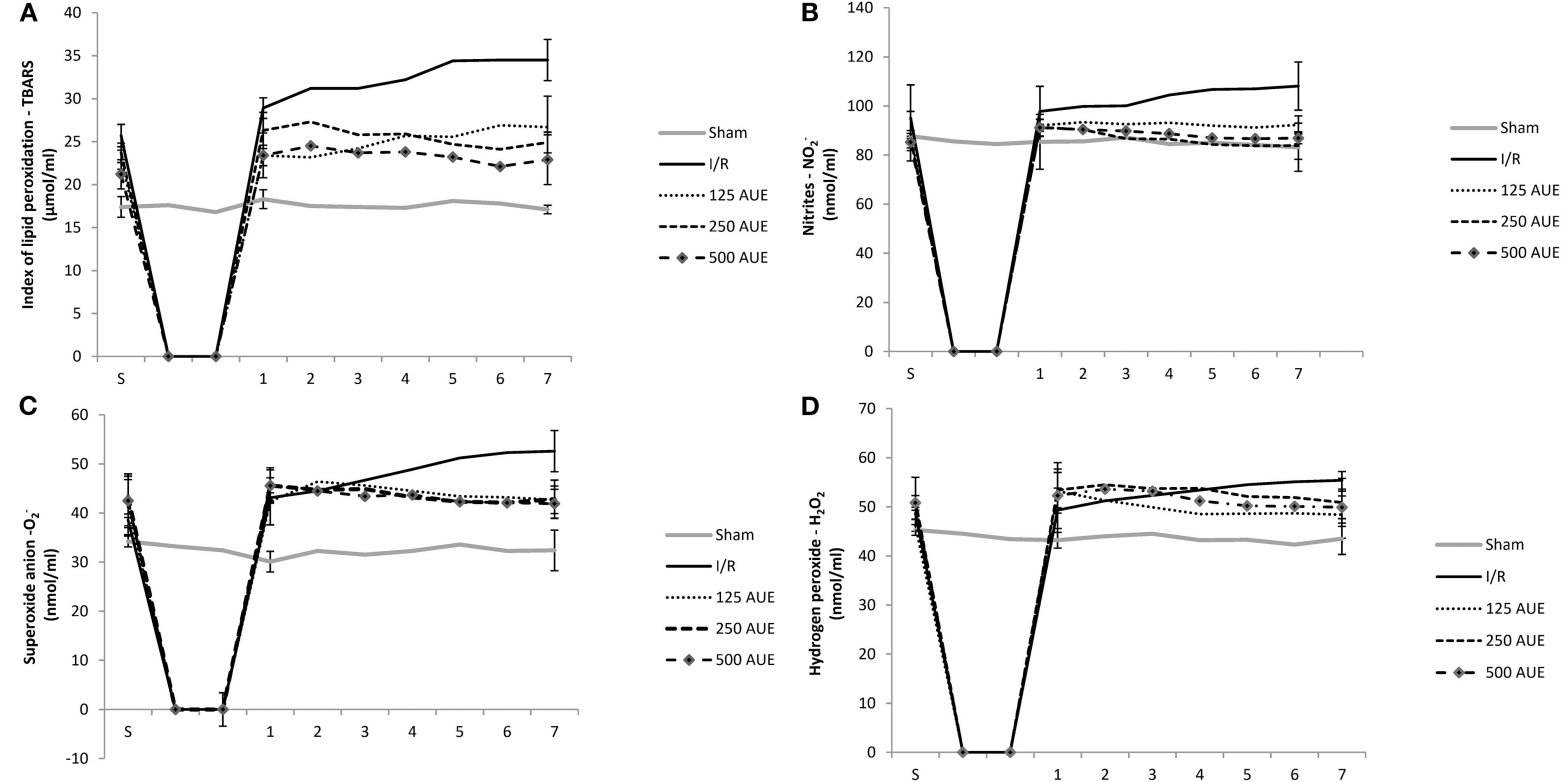
Effects of consumption of increasing doses of AUE on the value of pro-oxidative markers in coronary venous effluent measured as an index of lipid peroxidation (TBARS) **(A)**, nitrites (NO_2_^−^) **(B)**, superoxide anion **(C)**, hydrogen peroxide (H_2_O_2_) **(D)** (*n* = 50, 10 animals per group). Values are shown as mean ± SE.

The level of TBARS was significantly increased in the I/R group at all points of interest compared to the concentrations of these pro-oxidative markers in groups treated with AUE as well as in the sham group. A significantly higher TBARS value was observed after administration of the mean dose of extract compared to the remaining two doses in point 1. However, consumption of the highest dose of this methanol extract was associated with the lowest level of TBARS in the S period and other points (points 1–7) compared to two other experimental groups (Figure 2 and Table 2).

**Table 2:** Oxidative stress parameters from coronary venous effluent.

**Groups/**	**TBARS**	**O**_**2**_^**−**^ **(nmol/ml)**	**NO**_**2**_^**−**^ **(nmol/ml)**	**H**_**2**_O_**2**_**(nmol/ml)**
**parameters**	**(μmol/ml)**			
**Sham**				
S	17.4 ± 1.2^f^^−^^i^	34.2 ± 1.1	87.7 ± 10.1	45.3 ± 1.1
1	18.3 ± 1.1^f^^−^^i^	30.1 ± 2.1^f^^−^^i^	85.4 ± 11.2	43.2 ± 1.6^f^^−^^i^
7	17.1 ± 0.5^f^^−^^i^	32.4 ± 4.1^f^^−^^i^	83.2 ± 9.8	43.5 ± 3.2^f^^−^^i^
**I/R**				
S	25.7 ± 1.3^f^	95.2 ± 13.4	38.6 ± 1.2	48.7 ± 1.2
1	28.9 ± 1.2^f^	97.8 ± 10.2^f^	43.1 ± 1.2^f^	49.3 ± 4.5^f^
7	34.5 ± 2.4^a^^,^^f^	108.1 ± 9.8^a^^,^^b^^,^^f^	52.6 ± 4.4^a^^,^^b^^,^^f^	55.4 ± 1.88^a^^,^^b^^,^^f^
**AUE125**				
S	22.9 ± 1.11^g^	88.4 ± 1.6	41.2 ± 5.6	46.2 ± 1.2
1	23.4 ± 1.19^c^^,^^g^^*^	92.1 ± 2.4^g^	42.4 ± 4.8	53.2 ± 4.5^a^^g^
7	26.7 ± 3.7^a^^,^^d^^,^^g^^*^	92.3 ± 3.7^g^^*^	42.7 ± 2.8^g^^*^	48.4 ± 4.8^g^^*^
**AUE250**				
S	23.7 ± 1.4^h^	86.9 ± 3.5^*^	43.3 ± 2.6	51.2 ± 4.2
1	26.3 ± 2.7^c^^,^^e^^,^^h^	91.3 ± 2.2^h^	45.6 ± 3.8	53.4 ± 6.6^h^
7	24.9 ± 1.9^h^*	83.9 ± 3.9^h^*	42.8 ± 2.7^*^	50.9 ± 3.9^h^
**AUE500**				
S	21.2 ± 1.7^i^	85.3 ± 5.4	42.5 ± 5.9	50.8 ± 1.5
1	23.4 ± 2.6^e^^,^^i^	91.2 ± 3.9^i^	45.6 ± 2.9	52.3 ± 6.7^i^
7	22.9 ± 2.9^d^^,^^i^^*^	86.9 ± 6.7^i^^*^	41.9 ± 3.2^*^	49.9 ± 2.3^i^

*Mean values ± SE in three points of interest. S, stabilization; 1, first minute of reperfusion; 7, 30th min of reperfusion*;

* *Statistically significant difference at the level of p < 0.05 between the group I/R and AUE groups at the point of interest*;

a *Statistically significant difference at the level of p < 0.05 in relation to point S within the group*;

b *Statistically significant difference at the level of p < 0.05 in relation to the point 1 within the group*;

c *Statistically significant difference at the level of p < 0.05 between AUE125 and AUE250*;

d *Statistically significant difference at the level of p < 0.05 between AUE125 and AUE500*;

e *Statistically significant difference at the level of p < 0.05 between AUE250 and AUE500*

f *Statistically significant difference at the level of p < 0.05 between sham and I/R*;

g *Statistically significant difference at the level of p < 0.05 between sham and AUE125*;

h *Statistically significant difference at the level of p < 0.05 between sham and 250AUE*;

i *Statistically significant difference at the level of p < 0.05 between sham and AUE500*.

#### Level of NO_2_^–^

The level of this marker at the stabilization period did not differ significantly between all tested groups. However, after ischemia, the most prominent increase was noticed in the I/R group compared to the others. Administration of *A. ursinum* led to a significant drop in NO_2_^−^ value during points 1 and 7 as in S in comparison to the I/R group. However, concentrations of NO_2_^−^ did not vary significantly between groups treated with AUE in increasing doses (Figure 2 and Table 2).

#### Level of O_2_^–^

A significantly lower value of this pro-oxidative marker was observed in the sham group during points 1 and 7 compared to the others. The levels of O_2_ did not differ significantly within the experimental groups, while in the I/R group, an increase in production of this pro-oxidative marker was observed at the end point of the following period in comparison to values before ischemia. Additionally, it was found that *A. ursinum* consumed in any of three different doses led to a significant drop in O_2_ value compared to the I/R group (Figure 2 and Table 2).

#### Level of H_2_O_2_

At the end of the reperfusion period (point 7), an elevated level of H_2_O_2_ was observed in the I/R group compared to animals that drank AUE. Nevertheless, a significantly reduced level of H_2_O_2_ was observed in the sham group comparing to animals from I/R and AUE groups during points 1 and 7. However, there were no statistically significant differences in the value of H_2_O_2_ within groups treated with *A. ursinum* in increasing doses (Figure 2 and Table 2).

### Effects of *A. ursinum* Extract on Systemic Redox State

**Figure 3:**
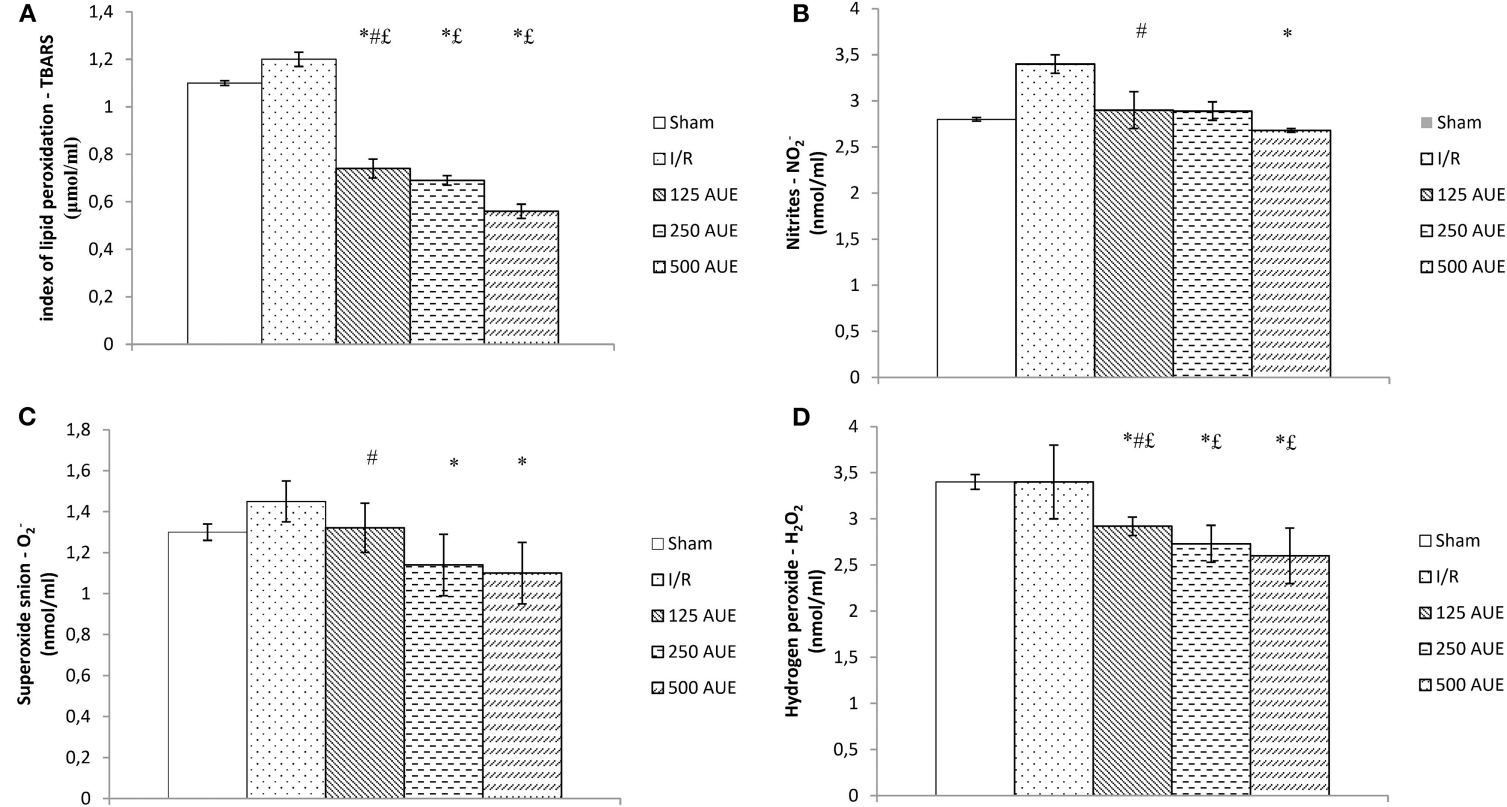
Effects of consumption of increasing doses of AUE on the value of pro-oxidative markers in plasma samples measured as index of lipid peroxidation (TBARS) **(A)**, NO_2_^−^ **(B)**, superoxide anion **(C)**, H_2_O_2_ **(D)** (*n* = 50, 10 animals per group). Values are shown as mean ± SE. ^*^Statistically significant difference at the level of *p* < 0.05 in relation to the I/R group; ^#^Statistically significant difference at the level of *p* < 0.05 compared to 500AUE;^£^Statistically significant difference at the level of *p* < 0.05 compared to sham. *n* = 10 animals per group.

All tested pro-oxidants were statistically significantly reduced in the group of rats using AUE at a dose of 500 mg/kg compared to the I/R group. Furthermore, pretreatment with AUE extracts in different doses led to remarkably decreased values of TBARS and H_2_O_2_ compared to the animals that were not exposed to the ischemic condition. However, there were no statistical differences in the values of NO_2_^−^ and O_2_^−^ between AUE groups and sham. Application of the extract at a dose of 250 mg/kg reduced the values of O_2_^−^, H_2_O_2_, and TBARS, while the lowest dose led to a decrease in the concentration of TBARS and H_2_O_2_ compared to untreated rats in the ischemic condition. By comparing the values of pro-oxidants between the groups on the treatment with different doses, lower values of all markers are observed after the treatment with the extract at a dose of 500 mg/kg in relation to 125 mg/kg (Figure 3).

**Figure 4:**
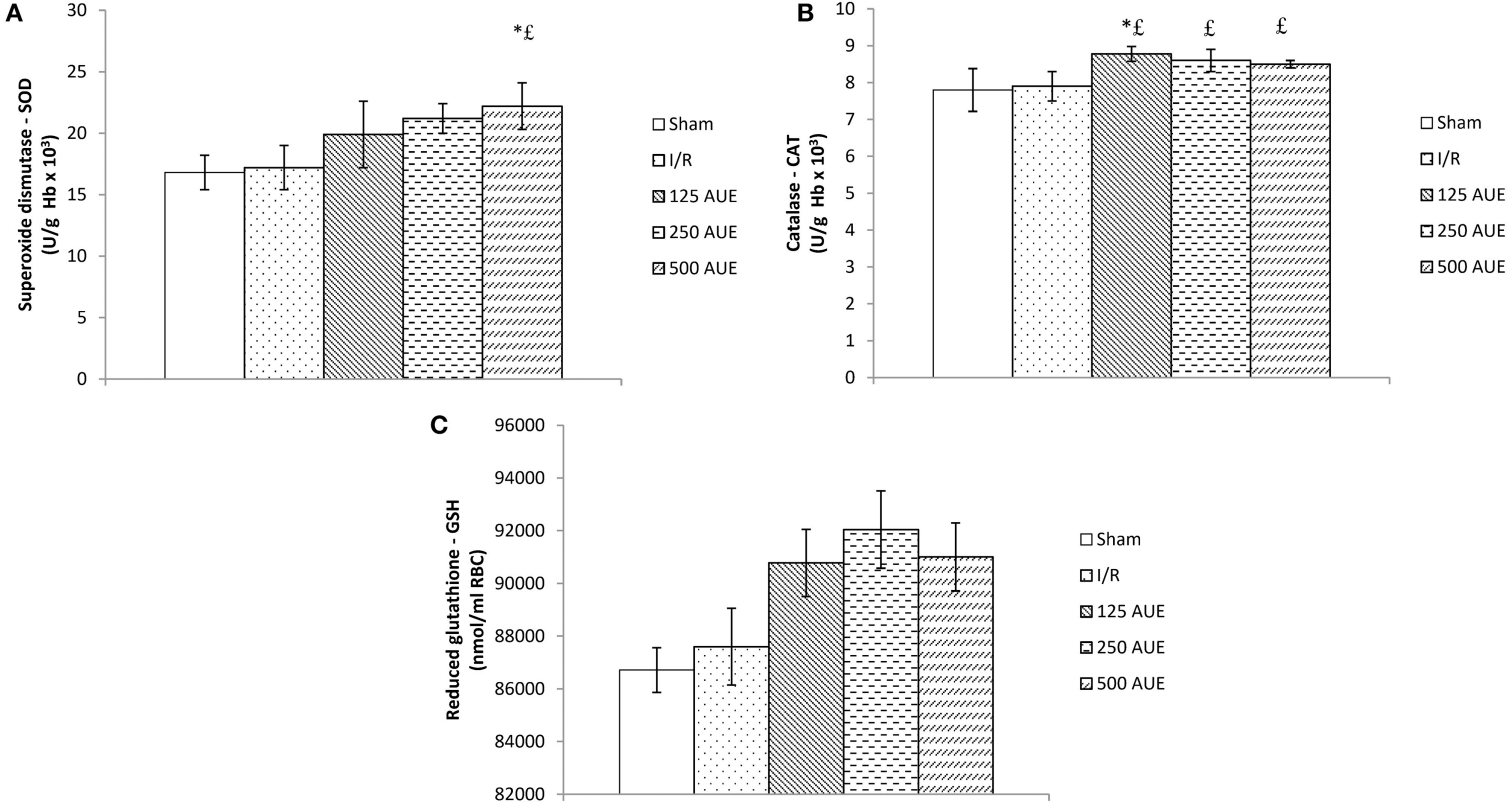
Effects of consumption of increasing doses of AUE on the value of superoxide dismutase (SOD) **(A)**, catalase (CAT) **(B)**, and reduced glutathione (GSH) **(C)** (n = 50, 10 animals per group). Values are shown as mean ± SE. ^*^Statistically significant difference at the level of *p* < 0.05 in relation to the I/R group;^£^Statistically significant difference at the level of *p* < 0.05 compared to sham. n = 10 animals per group.

The application of AUE caused a significant elevation in the antioxidant activity compared to I/R and sham rats. However, the highest dose of extract managed to lead to the highest SOD value compared to the I/R and sham groups, while the application in the lowest dose led to a significant jump in the CAT value. On the other hand, the GSH activity did not change significantly under the influence of AUE in all three doses (Figure 4).

### Effects of *A. ursinum* Extract on Tissue Antioxidant Defense System

**Figure 5:**
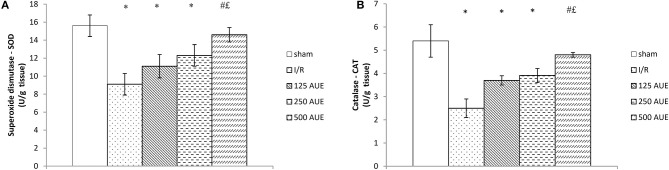
Effects of consumption of increasing doses of AUE on the value of antioxidant defense system in heart tissue measured as superoxide dismutase (SOD) **(A)**, catalase (CAT) **(B)** (*n* = 50, 10 animals per group). Values are shown as mean ± SE. ^*^Statistically significant difference at the level of *p* < 0.05 in relation to the sham group; ^#^Statistically significant difference at the level of *p* < 0.05 compared to I/R group;^£^Statistically significant difference at the level of *p* < 0.05 compared to sham.

The activities of SOD and CAT were significantly reduced in the I/R group compared to sham, but the consumption of AUE extract remarkably improved the values of these antioxidant markers. Also, we observed that the highest dose of the extract led to the most prominent increase of CAT and SOD in heart tissue compared to the two other AUE extract groups (Figure 5).

### Effects of *A. ursinum* Extract on Heart Morphology

**Figure 6:**
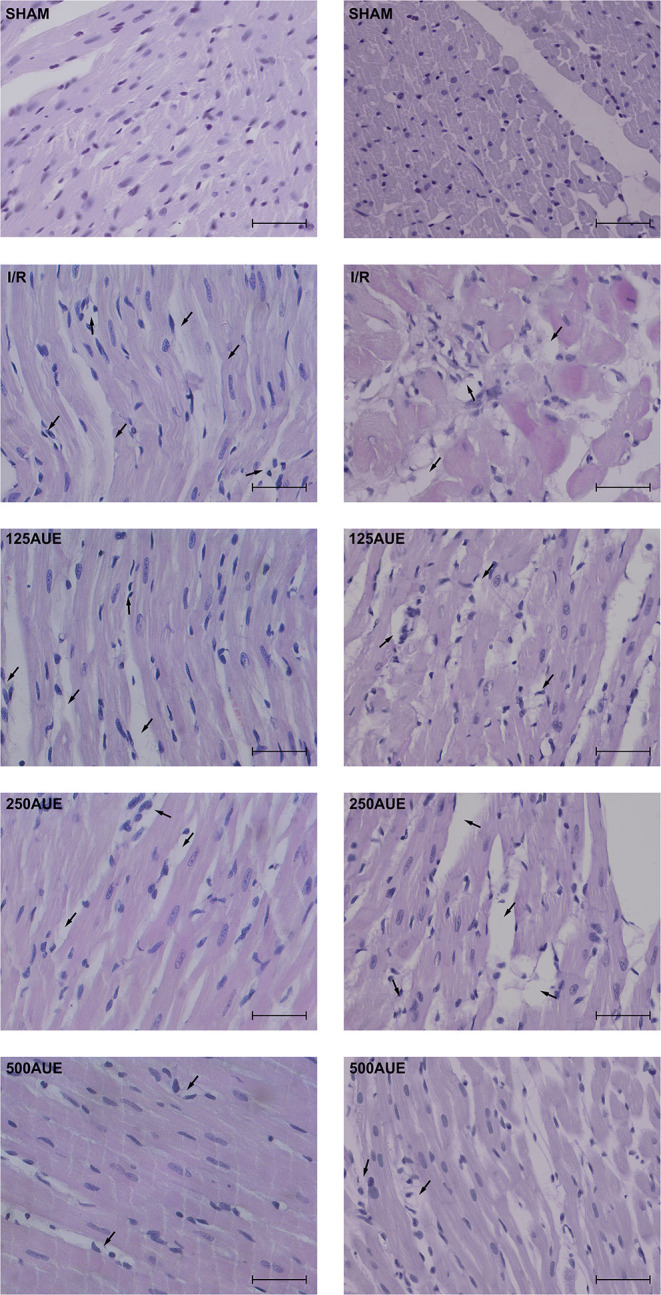
Representative photographs (magnification ×40; bar = 25μm) of hematoxylin and eosin staining of cardiac muscle tissue.

Microphotographs of the cardiac tissue in the sham control group showed regular morphological structure without degenerative changes and cellular infiltrate. Hypertrophy of individual muscle fibers with degenerative changes, hypercellularity (without cellular infiltrate), interstitial edema, and nuclear pyknosis were observed in the I/R group. However, in the group of animals treated with the lowest dose of *A. ursinum* hypertrophy of individual muscle fibers, degenerative changes, and hypercellularity were slightly less noticeable compared to the I/R group. Additionally, administration of AUE at a dose of 250 mg/kg led to less visible degenerative changes, hypercellularity, and interstitial edema compared to the I/R group and group treated with less dose of extract. The degree of degenerative changes and the presence of hypercellularity and interstitial edema were at least evident in the group that applied AUE at the highest dose but slightly altered nuclei were presented (Figure 6 and Table 3).

**Table 3:** Histological changes related to the degree of presence or absence of degenerative changes, dilated interstitium, and hypercellularity of cardiomyocytes.

**Groups/**	**Degenerative**	**Dilated**	**Hypercellularity**
**parameters**	**changes**	**interstitium**	
Sham	–	–	–
I/R	**+ + +**	**+**	**+ + +**
AUE125	**++**	**+**	**++**
AUE250	**++**	–	**++**
AUE500	**+**	–	**+**

## Discussion

It is well-recognized that cardiac dysfunction is often associated with elevated level of ROS or reactive nitrogen species (RNS) in both animal models of I/R injury and surgically induced myocardial infarction (MI) (Navarro-Yepes et al., 2014; Kurutas, 2016). Despite significant advances in the development of diagnostic and therapeutic strategies for patients with cardiovascular diseases (CVDs), the optimal algorithm is still not found. Additionally, the serious side effects of the available drugs limit their usage and still stayed as an important problem for the health system (Lovegrove et al., 2017; Khan et al., 2021). In that sense, more attention is being directed toward dietary intake of polyphenols as natural antioxidants due to their potential to modulate redox signaling thus providing cardioprotection (Das et al., 2015). Numerous preclinical (Kurian and Paddikkala, 2009, 2010; Kurian et al., 2010) and clinical studies have demonstrated that consumption of polyphenols, which are present in dietary plants such as *A. ursinum* is associated with cardiovascular risk reduction. The great therapeutic potential of polyphenols and extracts containing polyphenols is attributed to their antioxidant, immunomodulatory, anti-inflammatory, and vasodilatory activities. So far there is no enough information regarding the effects of *A. ursinum* on I/R injury, however, we hypothesized that secondary metabolites present in this plant species may help salvage myocardium exposed to ischemia. Therefore, the present study was undertaken to evaluate the effects of the chronic application of increasing doses of *A. ursinum* leaves extracts to support the heart to overcome stress conditions of ischemia and reperfusion.

The results of the study showed that 20-min ischemia followed by 30-min reperfusion was associated with lower absolute values of dp/dt max, dp/dt min, and SLVP at the end of the reperfusion period compared to the S and values in the sham group, indicating deterioration of contractility and relaxation force of the heart and systolic function. In line with the results, numerous studies indicated that the establishment of flow through tissue causes the accumulation of the Ca^2+^ and extracellular K^+^ which synergistically worsens the contractile strength of the heart (Jernryd et al., 2020). In addition, it was observed a reduction in CF at the last point of the reperfusion period in the I/R group, which further confirms the ability of reperfusion to paradoxically worsen heart function. On the other hand, treatment with the lowest dose of AUE did not change most of cardiodynamic parameters during the observed period, which in turn reflects the potential of methanol extract at a dose of 125 mg/kg to enable the heart to work smoothly in reperfusion. Additionally, the application of AUE at a dose of 250 mg/kg managed to prevent variations in the values of cardiac function parameters indicating a preserved myocardial response to I/R. The highest dose of wild garlic extract not only prevented depression of cardiac function but also coped to improve the contractile capacity of the heart, which was verified by significantly increased values of dp/dt min in the last minute of reperfusion in relation to S moment thus enabling myocardium to relax without fluctuations in force. Also, the values of almost all cardiodynamic parameters were higher in the group exposed to the highest dose of *A. ursinum* compared to the I/R ones reflecting the potential of AUE to improve inotropic and lusitropic properties of the heart (Figure 1). This is in line with the reports that administration of polyphenols or extracts containing polyphenols exert may help in the preservation of ventricular contractility after ischemia (Kanno et al., 2013). The observed values of cardiodynamic variables in the study indicate a significant role of *A. ursinum* in the heart preconditioning maneuver.

Cardioprotective effects of AUE observed in the study are in correlation with previously published studies. More than two decades ago it has been proven that a ramson-containing diet has the potential to minimize the size of the ischemia zone and exert benefits in I/R-induced arrhythmias (Rietz et al., 1993). The proposed mechanisms *via* AUE that achieves cardioprotection may be linked to its effect on the ACE enzyme, while the prostaglandin system is not responsible for the beneficial effects of this plant species. Moreover, it was observed that the lyophilisate of wild garlic leaves improved the function of the heart of an isolated rabbit with induced hypercholesterolemia, which was manifested through significantly higher post ischemic values of aortic flow compared to untreated rats. In this study, echocardiographic measurements suggest that diastolic function was improved in animals with hypercholesterolemia that consumed AUE. This statement is in correlation with the results which emphasize the effect of this plant on proper heart work since the relaxation phase determines the filling of the heart and end-heart work (Bombicz et al., 2016). Given the limited number of studies that have investigated the role of *A. ursinum* in the I/R of the heart, the results of the study can be explained based on chemical composition and compounds in these plants that are known to possess cardioprotective properties. The protective effects of polyphenols on cardiac contractile power can be explained by the effect on the Ca^2+^-ATP-ase SERCA pump. Due to the uptake of Ca^2+^ from the cytoplasm into the sarcoplasmic reticulum by the SERCA pump, Ca^2+^ overload occurs which contributes to cardiac dysfunction in the I/R conditions. In this sense, by acting on the SERCA pump, polyphenols accelerate the return of intracellular Ca^2+^ reserves, which provides a sufficient amount of this ion for the next contraction (Misquitta et al., 2002; Talukder et al., 2008; Garcia-Dorado et al., 2012). One more finding of the study can be explained by the action of polyphenols. Namely, daily intake of AUE led to preserved coronary response which is reflected by constant values of coronary flow. This can be attributed to the influence of polyphenols to increase NO bioavailability by activating endothelial constitutive NO synthase (eNOS) which seems to be decreased in ischemic conditions (Cebova and Pechanova, 2020).

In the second part of the study, we examined whether 4-week treatment with increasing doses of *A. ursinum* affects redox homeostasis and alters the production of pro-oxidants in coronary venous effluent. Results of the research had proved that I/R injury was related to increased oxidative stress which is reflected in a remarkably higher level of TBARS, NO_2_^−^, O_2_^−^, and H_2_O_2_ in coronary venous effluent at the early phase of reperfusion as well as at the end of the experimental period compared to S. Additionally, the level of all pro-oxidants was significantly higher in I/R compared to the sham group, thus, indicating disturbed redox status in ischemia conditions. On the other hand, administration of the lowest dose of AUE did not cause any fluctuation in the values of pro-oxidants during the observed period. Even more, treatment with the extract at a dose of 250 mg/kg managed not only to prevent fluctuations in pro-oxidants, but also led to a decrease of all pro-oxidative markers in relation to the S period. A similar trend was observed after the application of the highest dose of AUE, while at the end of the reperfusion, a significant decrease in TBARS, NO_2_^−^, and O_2_ was observed compared to the I/R group. In addition, pretreatment with AUE prevented the increase of O_2_^−^ and H_2_O_2_ as markers of myocardial oxidative damage, while also inhibited ischemia-induced lipid peroxidation in heart tissue, thus maintaining membrane integrity (Figure 2). Superoxide anion is primarily formed as an intermediate in biochemical reactions and compared with other free radicals has a relatively long half-life that enables diffusion within the cell. One of the proposed mechanisms through which polyphenols from AUE protect myocardium from O_2_^−^ induced oxidative damage involves inhibition of its source, such as xanthine oxidase and nicotinamide adenine dinucleotide phosphate-oxidase (NADPH) (Pisoschi and Pop, 2015). Moreover, literature data suggest that the antioxidant action of phenolics is based mainly on their direct free radical scavenging activity and metal chelating properties and on their effect on cell signaling pathways and gene expression (Liu et al., 2019). Although superoxide is generally considered relatively unreactive compared with other radical species, it reacts with NO giving a highly reactive, damaging nitrogen species, namely peroxynitrite (ONOO-), a powerful oxidant vs. many biological molecules (Pisoschi and Pop, 2015). Since NO is unstable and rapidly decomposed into stable NO_2_^−^, in the study, we indirectly measured levels of NO through NO_2_^−^ levels. Levels of NO_2_^−^ in the I/R group were significantly increased at the end of the reperfusion period in comparison to S, which is in correlation with the decreased coronary flow in these rats. Degradation of NO responsible for maintenance of coronary vasodilatory response is in agreement with elevated NO_2_^−^ levels (Bradic et al., 2019; Cebova and Pechanova, 2020). On the contrary, values of both NO_2_^−^ and CF remained constant in AUE treated groups and might explain the results. Additionally, studies have shown that garlic extract protects endothelial cells from H_2_O_2_-induced oxidative damage by inhibiting lipid peroxidation and increasing endogenous antioxidant defense systems (Yamasaki et al., 1994). Taking into account significant alleviation of cardiac oxidative stress noticed in all treated rats compared to untreated groups, it might be concluded that even the lowest dose was sufficient to provide a necessary reduction in the monitored pro-oxidant markers. To precisely reveal if the mechanism responsible for better effects of the higher dose of extraction myocardial functional and morphological parameters are independent of modulation of redox homeostasis, we performed an additional analysis of CAT and SOD activity in heart tissue. The results of the study illustrated that I/R injury was related to a significant drop of antioxidative enzymes activity while A. ursinum pretreatments significantly recovered the levels of SOD and CAT in heart tissue. Although both lower doses of AUE improved antioxidant tissue defense, the highest dose of extract achieved the most prominent increase of the CAT and SOD compared to the I/R group (Figure 5). Based on the findings in this study model, mechanisms of antioxidant action of AUE involve both direct scavenging activity and enhancing antioxidant enzymes activity, especially in a higher dose.

Another aspect of the study involved examining if AUE applied in different doses can change the systemic concentration of pro-oxidants. The results demonstrated a significant drop in the values of all markers after treatment with the extract compared to the I/R group but with the most prominent changes in groups treated with the highest dose. The same trend was observed in SOD activity, while when it comes to CAT activity, the lowest dose led to the most significant increase in relation to the I/R group. However, GSH levels did not change significantly after 4 weeks of intake of *A. ursinum* in all three doses (Figure 4). In a study by Masjedi et al. (2013), methanol extract of *A. ursinum* was applied in diabetic rats at doses of 60 and 120 mg/kg. The activity of SOD and CAT was significantly increased in treated animals compared to the I/R group, which is in accordance with the results, providing the ability of *A. ursinum* to reduce oxidative stress and provide beneficial systemic effects. A previously conducted study examined whether *A. ursinum* has better antioxidant properties compared to other Allium species. It was observed that wild garlic possesses a relatively higher concentration of antioxidants and better scavenger activity compared to the other Allium species from Balkan flora. The results of this study clearly indicated the ability of Allium species to remove free radicals and prevent oxidative stress from occurring (Stajner et al., 2008). According to a previously published study, methanolic extract was considered to be the richest in the number of phenolic acids and flavonoids due to its strongest ability (followed by ethanol) to isolate phenolic compounds (Ebrahim et al., 2013; Pavlović et al., 2017). Contrastingly, the results of the study conducted on cell lines that are previously exposed to doxorubicin treatment indicate the powerful antioxidant effect of ethanol AUE but poor the effect of the methanolic extract on the antioxidant capacity of wild garlic. The discrepancy between their results and the findings of other studies may be attributed to different study designs and measurement of the impact of A. ursinum on different parameters of redox status by using various tests (Pop et al., 2020).

During ischemia, there are changes in the heart that can cause dysfunction and necrosis (Moraes-Silva et al., 2017). However, reperfusion of the ischemic heart led to not only the functional deterioration of work but also the structural harmful changes that significantly disrupt the regular myocardial function. Histological results of the study showed that in untreated rats, I/R conditions led to degenerative changes with hypertrophy of individual muscle fibers. Previously, it was reported that ischemia increases microvascular permeability interstitial edema, impaired vasoregulation, inflammatory cell infiltration and dysfunction, and necrosis of parenchymal cells. On the other hand, the reperfusion leads to the creation and activation of various humoral mediators of inflammation and the formation of free radicals (Bradic et al., 2019). In the study, the best cardioprotective effect was observed in rats that consumed AUE at a dose of 500 mg/kg for 4 weeks, while hearts treated with lower doses were verified by similar morphological characteristics of the myocardium as in untreated rats (Figure 6).

The findings indicate the greater capacity of the highest dose to modulate antioxidant enzymes. Gradual increase in the dose of the applied extracts is followed by a more intensive impact of antioxidant enzymes and cardiac functional recovery, thus, suggesting that better protection of the cardiovascular system might be expected by daily consumption of higher doses of wild garlic.

## Conclusion

In conclusion, 4-week consumption of methanol extract of *A. ursinum* significantly improved recovery of myocardial function after I/R injury, which was partially mediated *via* a decrease in cardiac oxidative stress. However, further research in this field is certainly necessary for better understanding the overall therapeutic potential of this plant species in different models of myocardial ischemia-reperfusion injury. We speculate that the ability of AUE to prevent myocardial damage originates from additive and synergistic antioxidant activities of all present bioactive natural compounds.

## Data Availability Statement

The raw data supporting the conclusions of this article will be made available by the authors, without undue reservation.

## Ethics Statement

The animal study was reviewed and approved by Ethics committee for experimental animal well-being of the Faculty of Medical Sciences at the University of Kragujevac, Serbia.

## Author Contributions

MR and MK conducted the chronic treatment with *A. ursinum* and wrote the manuscript. JB, AP, and JJ performed experiments on the Langendorff apparatus. VZ, MK, and NJ designed the study and interpreted the results. SB and MT performed statistical analyses and assisted in the experiments. JS performed histopathological analyses. VJ and SB designed the study. All the authors contributed into the interpretation of results and to the writing of the manuscript.

## Conflict of Interest

The authors declare that the research was conducted in the absence of any commercial or financial relationships that could be construed as a potential conflict of interest.

## Publisher's Note

All claims expressed in this article are solely those of the authors and do not necessarily represent those of their affiliated organizations, or those of the publisher, the editors and the reviewers. Any product that may be evaluated in this article, or claim that may be made by its manufacturer, is not guaranteed or endorsed by the publisher.
