# Supplementary material for: New Insight Into the Cardioprotective Effects of Allium ursinum L. Extract Against Myocardial Ischemia-Reperfusion Injury
Source: Front Physiol. 2021 Jul 30;12:690696. doi: 10.3389/fphys.2021.690696 (PMC8361798; doi:10.3389/fphys.2021.690696)
Supplement: Supplementary file 1 [file Table_1.pdf]

**SUPPLEMENTARY TABLE 1. Histological changes related to the degree of presence or absence of degenerative changes, dilated interstitium and hypercellularity of cardiomyocytes.**

| Groups/Parametars | degenerative changes | dilated interstitium | hypercellularity |
|---|---|---|---|
| Sham | - | - | - |
| I/R | +++ | + | +++ |
| AUE125 | ++ | + | ++ |
| AUE250 | ++ | - | ++ |
| AUE500 | + | - | + |
