# Supplementary figures and images for: New Insight Into the Cardioprotective Effects of Allium ursinum L. Extract Against Myocardial Ischemia-Reperfusion Injury
Source: Front Physiol. 2021 Jul 30;12:690696. doi: 10.3389/fphys.2021.690696 (PMC8361798; doi:10.3389/fphys.2021.690696)

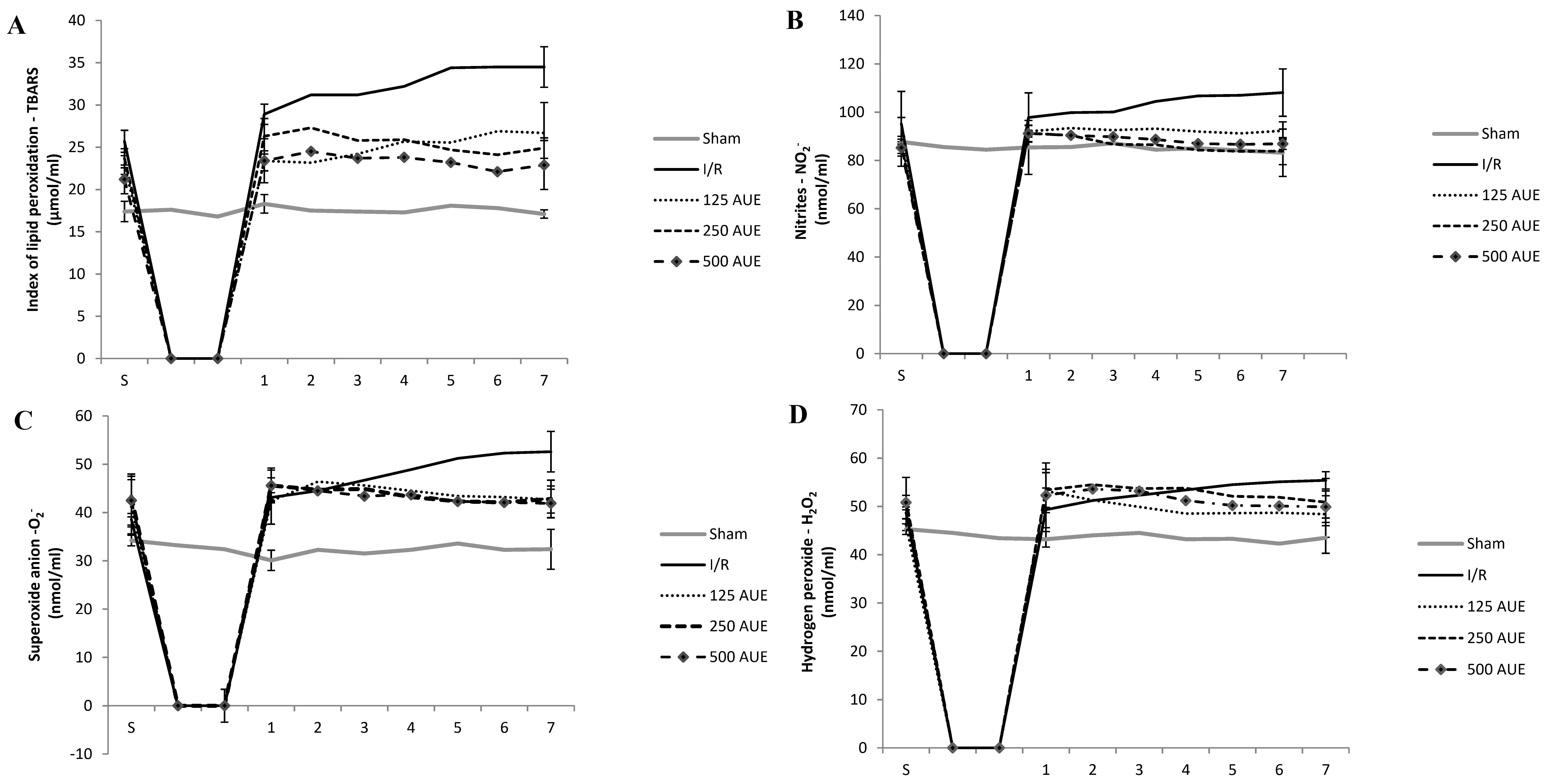

Supplement: Supplementary file 2 [file Image_1.tiff]
